# Effect of the change in antiviral therapy indication on identifying significant liver injury among chronic hepatitis B virus infections in the grey zone

**DOI:** 10.3389/fimmu.2022.1035923

**Published:** 2022-10-27

**Authors:** Shan Ren, Wenjing Wang, Junfeng Lu, Kefei Wang, Lina Ma, Yanhong Zheng, Sujun Zheng, Xinyue Chen

**Affiliations:** ^1^ First Department of Liver Disease Center, Beijing Youan Hospital, Capital Medical University, Beijing, China; ^2^ Beijing Institute of Hepatology Beijing Youan Hospital, Capital Medical University, Beijing, China

**Keywords:** chronic hepatitis B (CHB), grey zone (GZ), liver biopsy, significant liver injury, antiviral therapy, alanine aminotransferase (ALT), indication

## Abstract

**Objective:**

In clinical practice, a substantial proportion of chronic hepatitis B virus (HBV) infections that do not fit into any of the usual immune states are considered to be in the “grey zone (GZ)”. This study aimed to investigate the effect of the change in antiviral therapy indication on identifying significant hepatic injury among GZ patients.

**Methods:**

Patients with chronic HBV infections and a persistent normal alanine aminotransferase (ALT) level (PNALT) who underwent ultrasonography-guided percutaneous liver biopsy were examined retrospectively. Evidenced hepatic injury (EHI) was defined as an inflammation grade ≥2 (≥G2) and/or fibrosis stage ≥2 (≥F2). Complete clinical data, liver inflammation, and fibrosis grades were collected, and the levels of cytokines were detected by the Luminex technique, all of which were analysed to investigate the immune and histopathology states of the liver.

**Results:**

A total of 347 patients with chronic HBV infections and PNALT were categorized into immune tolerant (IT, n = 108), inactive HBV surface antigen (HBsAg) carrier (IHC, n = 61), GZ-1 (HBeAg positive in GZ, n = 92), and GZ-2 (HBeAg negative in GZ, n = 68) phases. Among them, 51.3% were in the GZ phase, and 50.1% presented with EHI. The IL-6 levels were higher in the EHI group than in the non-EHI group (2.77 *vs*. 1.53 pg/ml, Z = −13.32, p = 0.028). The monocyte chemoattractant protein 1 (MCP-1) level was positively correlated with HBV DNA (R = 0.64, p < 0.001) and HBeAg (R = 0.5, p < 0.001) but negatively correlated with fibrosis grade (R = −0.26, p = 0.048). The ratio of EHI in the GZ phase was 60.55%, which was significantly higher than that in patients in the IT (39.8%) and IHC phases (37.7%) (χ^2^ = 10.4, p = 0.006). A total of 46.69% of all patients exceeded the new ALT antiviral treatment threshold (30 U/L for men and 19 U/L for women). The EHI values in the IT and IHC phases below the new ALT threshold were 32.6% and 37.8%, respectively, whereas higher EHI values of 67.4% and 68.4% were seen in GZ-1 and GZ-2 patients, respectively, exceeding the new ALT threshold, and the difference was statistically significant (χ^2^ = 11.13, p < 0.001; χ^2^ = 14.22, p = 0.002). The median age in our cohort was 38.91 years, and only 21.03% were less than 30 years old. The EHI values in the IT and IHC patients <30 years old were 32.4% and 35.8%, respectively, while the ratio of EHI increased to 43.2% once patients were older than 30 years but still in the IT and IHC stages.

**Conclusion:**

Setting 30 years old as a cut-off and lowering the ALT threshold could facilitate screening for the presence of significant liver injury, especially for GZ patients. IL-6 was a good indicator of EHI, and MCP-1 was significantly positively correlated with HBV DNA but negatively correlated with liver fibrosis.

## Introduction

Chronic hepatitis B virus (HBV) infection is a public health problem, with approximately 257 million cases worldwide (1) and 70 million chronic HBV infections in China (2). Chronic HBV infection is a dynamic process that reflects the interaction between HBV replication and the host immune response. Investigators from Taiwan and Hong Kong, China, have introduced the concept of host immunity, and after several revisions of the paradigm, the natural history of chronic HBV infection is currently stratified into four phases: immune tolerance (IT, HBeAg positive with a high HBV DNA load), immune clearance (IC), inactive HBsAg carrier (IHC; HBeAg negative with HBV DNA <2,000 IU/ml), and reactivation (3–5); this has been widely adopted by guidelines (6–9). Antiviral therapy is recommended for patients within the IC and reactive phases due to elevated alanine aminotransferase (ALT), while IT and IHC patients with normal ALT levels could skip antivirals for the time being, as their inflammation/fibrosis in the liver tissue is not significant.

However, in clinical practice, some patients who are not categorized into any of the above four phases are considered to be in the “indeterminate phase” (10) or “grey zone (GZ)”. Especially in cases of normal ALT, HBeAg-positive patients may have a mild HBV DNA load, and HBeAg-negative patients can have HBV DNA ≥2,000 IU/ml. Therefore, the threshold of ALT and the level of HBV DNA are critical for distinguishing chronic infection from chronic hepatitis. Recently, “The Chronic Hepatitis B Treatment Algorithm” (11) in the United States recommended that regardless of HBeAg positivity or negativity, any case with elevated ALT (>30 U/L for men and >19 U/L for women) and HBV DNA ≥2,000 IU/ml should receive antiviral therapy. Subsequently, “The Expert Opinion on Expanding Antiviral Therapy for Chronic Hepatitis B” (12) in China adopted the same ALT threshold but lowered the indication further: once positive for HBV DNA [lower limit of detection (LLV) = 10–20 IU/ml] with elevated ALT, antiviral therapy was recommended.

Increasing evidence (13) of a benefit from antiviral therapy for patients outside the current criteria, as well as increasing accessibility and affordability of highly effective, low-drug-resistant drugs, makes it feasible to expand the indications for antiviral therapy. However, the effect of the change in antiviral therapy indication on identifying significant liver injury in the GZ phase remains uncertain. Therefore, we aimed to establish a retrospective cohort of chronic HBV infections with normal ALT levels who underwent ultrasonography-guided percutaneous liver biopsy. Complete clinical data, liver inflammation, and fibrosis grades were collected to investigate the effect of an expansion in indications of antiviral therapy on identifying significant liver injury among GZ patients and to select appropriate antiviral therapy (ART) strategies to maximize the benefits of treatment for the patients.

## Methods

### Study population

**Figure 1:**
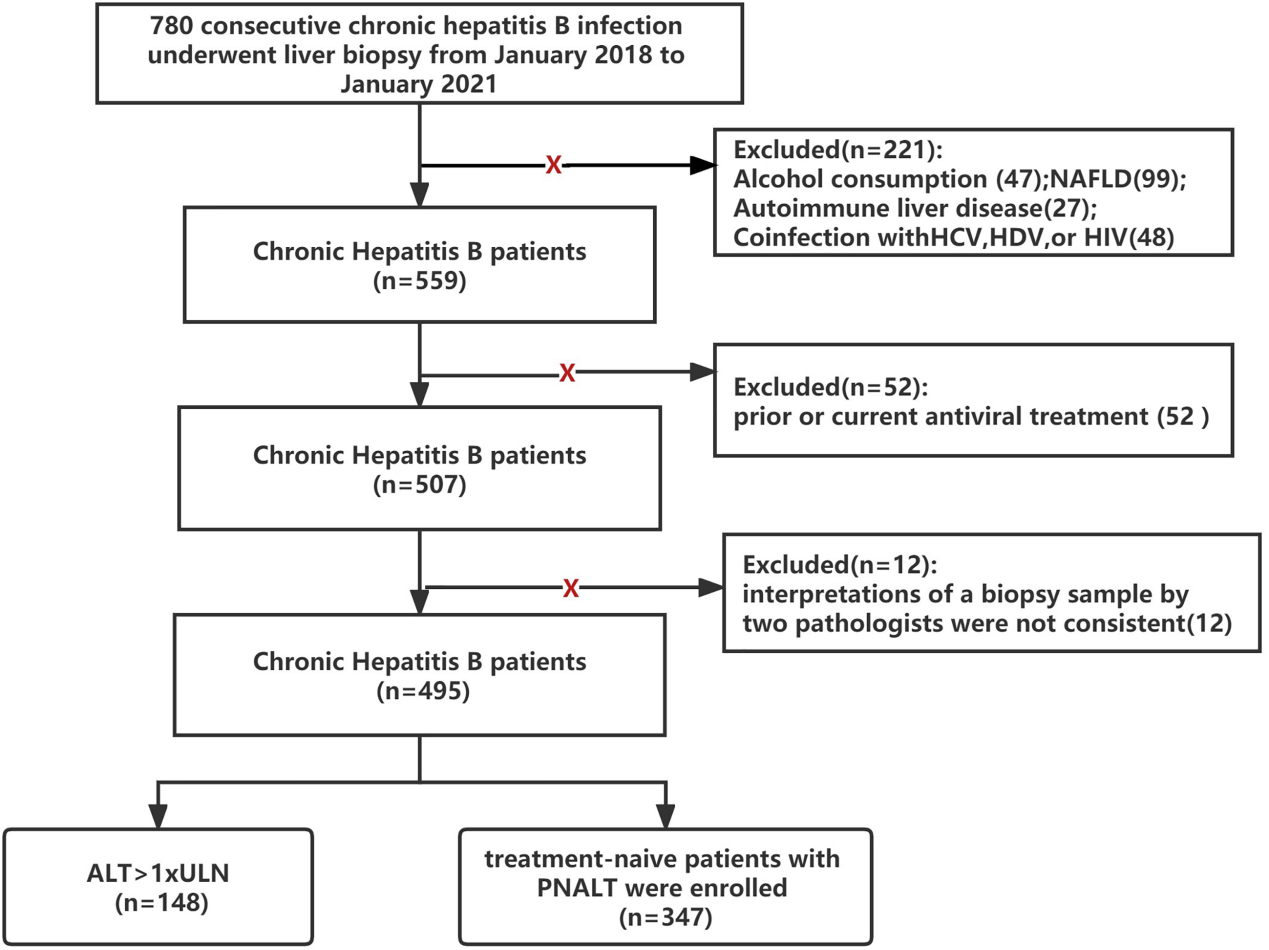
Flowchart of the study design (NAFLD, nonalcoholic fatty liver disease; HCV, hepatitis C virus; HDV, hepatitis D virus; HIV, human immunodeficiency virus; PNALT, persistent normal ALT; ULN, <40 U/L).

A total of 780 patients with chronic HBV infections who underwent liver biopsy at Beijing You’an Hospital, Capital Medical University (Beijing, China), from January 2018 to September 2021 were enrolled in this retrospective study. Chronic HBV infection was defined as the persistent presence of serum HBsAg for >6 months (6). Persistent normal ALT level (PNALT) was defined as normal ALT (upper limit of normal (ULN): ≦40 U/L) measured on at least two to three occasions at intervals of 3 months over 12 months. Patients with the following conditions were excluded: alcohol consumption >20 g/day (47 patients); accompanied by non-alcoholic fatty liver disease (99 patients); accompanied by autoimmune liver disease (27 patients); coinfection with hepatitis C virus, hepatitis D virus, or human immunodeficiency virus (48 patients); prior or current antiviral treatment (52 patients); ALT > 1 × ULN (148 patients); and the interpretations of a biopsy sample by two pathologists were not consistent (12 patients). Finally, 347 patients with chronic HBV infection and PNALT levels were included in this study (**Figure 1**).

The study protocol was approved by the Beijing You’an Hospital, Capital Medical ethics committee and was conducted in accordance with the 1975 Declaration of Helsinki and revised in 1983 (The Code of Ethics Approval (2020):089). The study had registered at clinicaltrials.gov (ID: ChiCTR2000040971, http://www.chictr.org.cn/com/25/edit.aspx?pid=65556&htm=4)

### Liver histological examination

Ultrasonography-guided percutaneous liver biopsy was performed using a 16-G disposable needle (Hepafix, B. Braun, Melsungen, Germany) under local anaesthesia. Liver tissue samples of minimum length (15 mm) or larger were immediately fixed with 10% formalin and embedded in paraffin. Liver tissue with at least six portal tracts was sufficient for histological scoring. The METAVIR scoring system was adopted as the diagnostic standard for liver inflammation and fibrosis according to the recommended Chinese guidelines on HBV prevention and treatment (2019 version) (6). The histological grading of liver inflammation was classified into G0–G4, and fibrosis was staged at F0–F4. Evidence of hepatic injury (EHI) was defined as an inflammation grade ≥2 (≥G2) and/or fibrosis stage ≥2 (≥F2). All of the biopsy samples were interpreted independently by at least two liver pathologists. If the interpretations of a biopsy sample by two pathologists were not consistent, we discarded the data (**Figure 1**).

### Laboratory tests

Fasting blood samples were obtained, and routine laboratory tests were performed at the same time as the liver biopsies. The serological markers of HBV were detected using commercially available enzyme-linked immunosorbent assay (ELISA) kits (ARCHITECT i2000 SR; Abbott, Wiesbaden, Germany). The serum biochemical parameters, including ALT, aspartate transaminase (AST), alkaline phosphatase (ALP), gamma-glutamyl transpeptidase (GGT), total bilirubin, albumin, and globulin, were measured on an automated biochemistry analyser (7600 Series; Hitachi, Tokyo, Japan). Routine blood test results, including white blood cell (WBC) count and platelet count, were evaluated on an automated haematology analyser (XT-2000i, Sysmex, Kobe, Japan). HBV DNA was quantified on a real-time polymerase chain reaction (PCR) system (ABI 7500; Applied Biosystems, Foster City, CA, USA) with the lowest detection limit at 20 IU/ml.

### Cytokines detected with Luminex kits

Each serum sample was analysed for cytokines, including monocyte chemoattractant protein 1 (MCP-1), soluble CD40 L (sCD40 L), fibroblast growth factor 2 (FGF2), interferon α (IFN‐α), interferon gamma (IFN‐γ), interleukin‐1 (IL‐1), IL-2, IL-6, IL-8, IL-10, IL‐17, IL-21, tumour necrosis factor‐α (TNF‐α), interferon-γ-inducible protein 10 (IP-10), and vascular endothelial growth factor-α (VEGF‐α) by Luminex bead-based MILLIPLEX^®^ assays using the Human Cytokine Panel A Magnetic Bead Panel kit (Millipore, Billerica, MA, USA) with the FlexMAP3D (Luminex) platform, and the cytokine level data were analysed using xPONENT^®^ software following the manufacturer’s instructions (14).

### Statistical analysis

The normality of all data was assessed using the Kolmogorov–Smirnov test. The baseline characteristics of the enrolled patients are as follows: normally distributed data are presented as the mean ± standard deviation, non-normally distributed continuous data are given as the median [interquartile range (IQR)], and categorical variables are given as the number (percentage). Chi-square tests (for categorical variables), Mann–Whitney tests (for non-normally distributed continuous variables), and t-tests (for normally distributed variables) were conducted to identify the significant differences between groups. Univariate and multiple logistic regression analyses were performed to identify the independent predictors of EHI. All significance tests were two-tailed, and p < 0.05 was considered statistically significant. All statistical analyses were carried out using SPSS statistical software version 23.0 (SPSS Inc., Chicago, IL, USA).

## Results

### Baseline characteristics of the enrolled patients

The baseline characteristics of 347 chronic HBV infections with PNALT are summarized in **Table 1**. The majority of the enrolled patients were men (210, 60.5%), HBeAg-positive (200, 57.64%), and middle-aged [38.91 (16–66) years], and 51.3% were in the GZ phase. The distribution of their different immune statuses was as follows based on the HBV Guidelines in China (2019 version) (8): IT (HBeAg-positive, HBV DNA >2 × 10^7^ IU/ml), 108 (31.12%) patients; GZ-1 (HBeAg-positive with HBV DNA ≦2 × 10^7^ IU/ml), 92 (26.52%) patients; GZ-2 (HBeAg-negative, HBV DNA >2,000 IU/ml), 86 (24.78%) patients; and IHC (HBeAg-negative, HBV DNA ≦2,000 IU/ml), 61 (17.58%) patients. The patients with HBeAg-negative HBV infections were older **Figure 3C**, especially the GZ-2 patients (median, 43.5 *vs*. 34.5–40 years, p < 0.001). A significantly higher ALT level was observed in HBeAg-positive patients than in HBeAg-negative patients (28.36 ± 9.49 *vs*. 23.59 ± 9.62 U/L, p < 0.001).

**Table 1:** Baseline characteristics of enrolled patients.

Clinical characteristics	Total (n = 347)	HBeAg-positive patients (n = 200, 57.64%)		HBeAg-negative patients (n = 147, 42.36%)		Value
		IT (n = 108)	GZ-1 (n = 92)	GZ-2 (n = 86)	IHC (n = 61)	
Age (years)	38.91 (16–66)	34.5 (19–54)	37.63 (16–61)	43.5 (23–66)	40 (24–61)	Z = 39.78, p < 0.001
Male, n (%)	210 (60.5%)	63 (58.3%)	65 (70.65%)	43 (50%)	39 (63.9%)	*X* ^2^ = 8.45, p = 0.038
BMI (kg/m^2^)	22.82 ± 4.23	21.42 ± 4.23	23.1 ± 3.92	22.32 ± 5.12	20.11 ± 4.12	F = 0.28, p = 0.34
ALT (U/L)	26.34 ± 9.82	28.64 ± 9.38	28.03 ± 9.66	24.68 ± 9.90	22.07 ± 9.07	F = 8.02, p < 0.001
AST (U/L)	23.21 ± 11.24	22.10 ± 10.12	26.82 ± 15.46	23.14 ± 8.81	22.07 ± 9.07	F = 8.02, p < 0.001
PLT (10^9^/L)	206 ± 78.82	248 ± 96.00	218 ± 86.00	185 ± 62.00	209 ± 89.00	F = 0.83, p = 0.09
ALB (g/L)	43.0 ± 4.20	45.0 ± 5.17	43.0 ± 3.65	44.0 ± 3.78	41.32 ± 5.16	F = 0.78, p = 0.64
ALP (U/L)	70.0 ± 29.0	72.0 ± 28.0	73.2 ± 23.4	76.0 ± 21.8	71.0 ± 28.0	F = 0.66, p = 0.61
GGT (U/L)	22.0 ± 27.0	20.0 ± 13.0	24.0 ± 18.0	20.9 ± 14.8	23.4 ± 17.0	F = 0.59, p = 0.56
Genotype C (n, %)	266 (76.1%)	82 (75.92%)	69 (75%)	34 (77.27%)	84 (81.53%)	*χ* ^2^ = 3.46, p = 0.09
HBV DNA (log10 IU/ml)	5.40 ± 2.25	8.14 ± 0.60	4.99 ± 1.47	4.40 ± 1.03	2.57 ± 0.56	F = 655.4, p < 0.001
HBsAg (log10 IU/ml)	3.55 ± 0.94	4.07 ± 0.77	3.58 ± 0.74	3.23 ± 0.65	2.86 ± 1.22	F = 22.78, p < 0.001
HBeAg (COI/ml)	565.49 ± 826	1,386.68 ± 703.65	506.97 ± 834.9	0.13 ± 0.15	0.11 ± 0.04	F = 92.51, p < 0.001
FibroScan:LSM (kPa)	6.14 ± 4.02	6.10 ± 6.35	6.42 ± 2.72	6.46 ± 2.38	5.31 ± 1.04	F = 1.21, p = 0.31
Liver histopathology						
Inflammation (G0–1 *vs*. G2–4)	233 *vs*. 114 (67.1 *vs*. 32.9)	75 *vs*. 33 (69.4 *vs*. 30.6)	49 *vs*. 43 (53.3 *vs*. 46.7)	59 *vs*. 27 (68.6 *vs*. 31.4)	50 *vs*. 11 (82 *vs*. 18)	*χ* ^2^ = 14.45, p = 0.002
Fibrosis (F0-1 *vs*. F2-4)	219 *vs*. 128 (63.1 *vs*. 36.9)	83 *vs.* 25 (76.9 *vs*. 23.1)	46 *vs*. 46 (50 *vs*. 50)	46 *vs*. 40 (53.5 *vs*. 46.5)	44 *vs*. 17 (72.1 *vs*. 27.9)	*χ* ^2^ = 21, p < 0.001
EHI *vs*. non-EHI	174 *vs*. 173 (50.1 *vs*. 49.9)	43 *vs*. 65 (39.8 *vs*. 60.2)	58 *vs*. 34 (63 *vs*. 37)	50 *vs*. 36 (58.1 *vs*. 41.9)	23 *vs*. 38 (37.7 *vs*. 62.3)	*χ* ^2^ = 16.7, p = 0.001

BMI, body mass index; PLT, platelet count; ALB, albumin; ALP, alkaline phosphatase; GGT, g-glutamyl transferase; evidenced hepatic injury (EHI) was defined as inflammation grade ≥2 (≥G2) and/or fibrosis stage ≥2 (≥F2).

**Figure 2:**
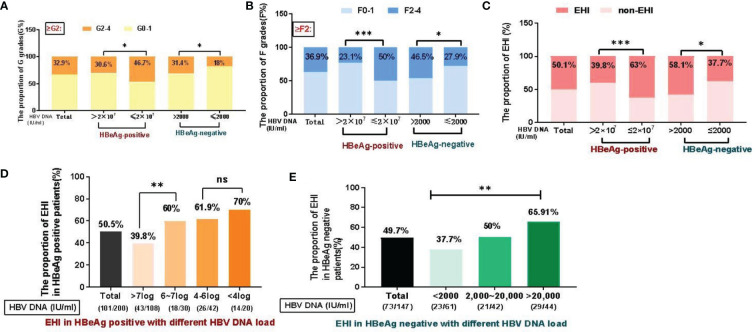
Distribution of liver inflammation and fibrosis in different immune status groups (%). **(A)** Significant differences in liver inflammation were observed among the groups. **(B)** Significant differences in liver fibrosis were observed among the groups. **(C)** EHI in different immune phases. **(D)** In the HBeAg-positive patient cohort, the overall proportion of EHI was 50.5% (101/200), 39.8% of patients with >7 log DNA load had EHI (43/108), followed by a decreased HBV DNA load, and 60–70% of subjects (18/30, 26/42, 14/20) had EHI (X^2^=11.2, P=0.01). **(E)** For HBeAg-negative patients, the overall proportion of EHI was 49.7% (73/147). The proportion of EHI in IHC (HBV DNA<2000 IU/ml) was significantly lower (37.7%) than that in patients with a higher HBV DNA load (50%~77.3%, X2=10.4, P=0.02). *p <0.05, **p <0.01, ***p <0.001; ns, not significant.

The histopathology data of the liver biopsy samples are shown in **Table 1** and **Figure 2**. A total of 32.9% of patients had G2–4 disease. Among these patients, GZ-1 patients had a significantly higher rate of G2–4 than IT patients (46.7% *vs*. 30.6%, χ^2^ = 5.52, p = 0.019), while GZ-2 patients had a significantly higher rate of G2–4 than IHC patients (31.4% *vs*. 18%, χ^2^ = 3.32, p = 0.049). In the case of liver fibrosis, GZ-1 patients had a significantly higher rate of F2–4 than IT patients (50% *vs*. 23.1%, χ^2^ = 15.64, p < 0.001), while GZ-2 patients had a significantly higher rate of F2–4 than IHC patients (46.5% *vs*. 27.9%, χ^2^ = 5.23, p = 0.049). Among all patients, 50.1% had an EHI ≥G2 and/or ≥F2. Typically, GZ patients had a higher rate of EHI, including GZ-1 (GZ-1: 63% *vs*. IT: 39.8%, χ^2^ = 10.72, p = 0.001) and GZ-2 (GZ-2: 58.1% *vs*. IHC: 37.7%, χ^2^ = 5.96, p = 0.015). Univariate analysis indicated that ALT, AST, HBV DNA, HBeAg, HBsAg, and FibroScan were associated with EHI. However, multiple logistic regression analysis indicated that HBsAg and FibroScan were associated with EHI in HBeAg-positive patients and that AST, HBsAg, and FibroScan were associated with EHI in HBeAg-negative patients (**Supplementary Table 1**).

In the HBeAg-positive cohort (n = 200), the overall proportion with EHI was 50.5% (101/200). Based on their different levels of HBV DNA load, the subjects were stratified into four groups, namely, >7 log (54%, 108/200), 6–7 log (15%, 30/200), 4–6 log (21%, 42/200), and <4 log (10%, 20/200), and liver biopsies with EHI in the different groups are depicted in **Figure 2D**: 39.8% of patients with >7 log DNA load had EHI (43/108), and in order of their HBV DNA load decreasing, 60% (18/30), 61.9% (26/42) and 70% (14/20) of the subjects had EHI, respectively (χ^2^ = 11.2, p = 0.01). For the HBeAg-negative cohort (n = 147), the overall ratio of EHI was 49.7% (73/147). Based on the different levels of HBV DNA load, the subjects were stratified into three groups, namely, <2,000 (41.5%, 61/147), 2,000–20,000 (28.6%, 42/147), and >20,000 (29.9%, 44/147). The EHI rate in IHC (HBV DNA <2,000 IU/ml) was significantly lower (39.8%) than that in 2,000–20,000 (50%) and >20,000 (65.91%) patients (χ^2^ = 10.4, p = 0.02).

### Effect of the change in antiviral therapy indication on identifying evidenced hepatic injury

#### New alanine aminotransferase treatment thresholds for antiviral therapy

**Figure 3:**
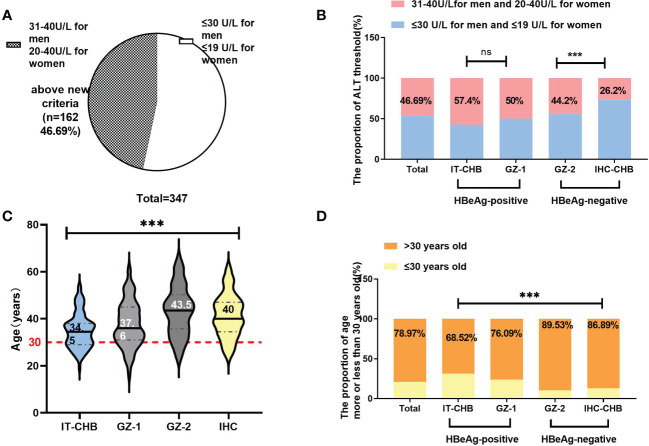
The change in antiviral therapy indication by ALT and age. **(A)** Among patients with ALT<40 U/L, only 53.3% were under the new threshold of ALT (30 U/L for men and 19 U/L for women). **(B)** Comparison of the proportion of patients exceeding the ALT threshold in different groups. **(C)** Comparison of the age in different groups (median); **(D)** Overall, 78.97% (274/347) of patients were over 30 years old. Comparison of the proportion of patients older than 30 years in different groups. ***p<0.001; ns: not significant.

Based on the new recommendations for the ALT antiviral treatment threshold (30 U/L for men and 19 U/L for women) (7), we further evaluated the ratio of EHI in different groups. Among the 347 chronic HBV infections, nearly half of them (162/347, 46.69%) were above this new threshold **Figure 3A**. Among these 162 patients, 56.2% (91/162) had EHI, which was significantly higher than that among the other patients (44.9%, 83/185), and was less than the new threshold (χ^2^ = 4.42, p = 0.036). In total, the proportion of patients with ALT ≤ 40 U/L who required antiviral therapy was 70.61% [(162 + 83)/347] according to the “Expert Opinion on Expanding Anti-HBV treatment” (7) in China.

In the HBeAg-positive cohort, 57.4% (62/108) of IT and 50% (46/92) of GZ-1 patients were above this new ALT threshold, and the EHI ratios were 45.2% and 67.4%, respectively **Figure 3B**. Among the patients below the ALT threshold, the ratio of EHI in GZ-1 was significantly higher than that in the IT group (58.7% *vs*. 32.6%, χ^2^ = 6.72, p = 0.01). However, there was no significant difference in the proportion of EHI between GZ-1 patients above the new ALT threshold and below it (67.4% *vs*. 58.7%, χ^2^ = 1.12, p = 0.34), and both were relatively high. It is worth noting that the ratio of EHI in IT patients with ≦”normal” ALT levels was significantly lower than that among GZ-1 patients >ALT threshold (32.6% *vs*. 67.4%, χ^2^ = 11.13, p < 0.001). The former was in the “truly” IT phase because of a high HBV DNA load (>2 × 10^7^ IU/ml) and quite low ALT levels (≤30 U/L for men and 19 U/L for women).

More significant discrepancies were seen between IHC and GZ-2 in the HBeAg-negative cohort. The vast majority (73.8%, 45/61) of IHC patients were within the new ALT threshold, and the ratio of EHI was comparable regardless of whether they exceeded or were under the new ALT threshold (37.5% *vs*. 37.8%, χ^2^ = 0.61, p = 0.87). In contrast, GZ-2 patients had a significantly higher EHI of 68.7% because of HBV DNA >2,000 IU/ml and ALT >new threshold (χ^2^ = 14.22, p = 0.002).

#### Age is a critical factor in identifying evidenced hepatic injury

**Figure 4:**
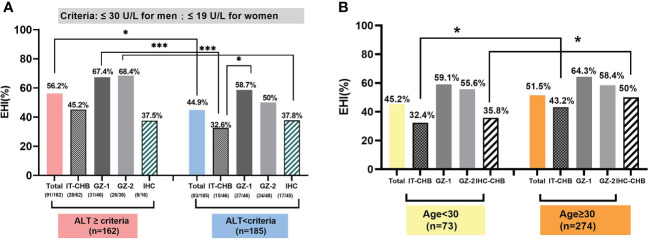
The effect of the change in antiviral therapy indication for age and ALT threshold in identifying EHI among those with chronic HBV infections. **(A)** Based on the new ALT threshold, IT and IHC patients presented a low EHI. **(B)** The proportion of EHI in patients more than 30 years old was 51.5%. Comparison of the proportion of EHI in each group, based on 30 years as the cut-off value.*p <0.05, ***p <0.001.

The median age in our cohort was 38.91 years, and 78.97% (274/347) of them were >30 years old. Among them, almost 90% of HBeAg-negative patients, including 89.53% of GZ-2 and 86.89% of IHC, were >30 years old **Figure 3D**, and the ratio was significantly higher than that in HBeAg-positive patients (IT: 68.52%, GZ-1: 76.09%, χ^2^ = 15.64, p = 0.001). In total, the ratio of EHI in patients >30 years old was 51.5% **Figure 4B**, and there was no significant difference in the ratio of EHI among GZ patients regardless of whether they were older or younger than 30 (55.6%–64.3%, χ^2^ = 1.34, p = 0.29). The ratio of EHI in the IT group was 32.4% for those <30 years old, while it increased to 43.2% for those >30 years old even though they were still in the IT stage (χ^2^ = 4.65, p = 0.04). A similar difference was observed in the IHC group, and the ratio of EHI was 35.8% in IHC patients <30 years old and increased to 50% when they were >30 years old (χ^2^ = 5.72, p = 0.03).

#### Correlation of cytokines with clinical characteristics and evidenced hepatic injury

**Figure 5:**
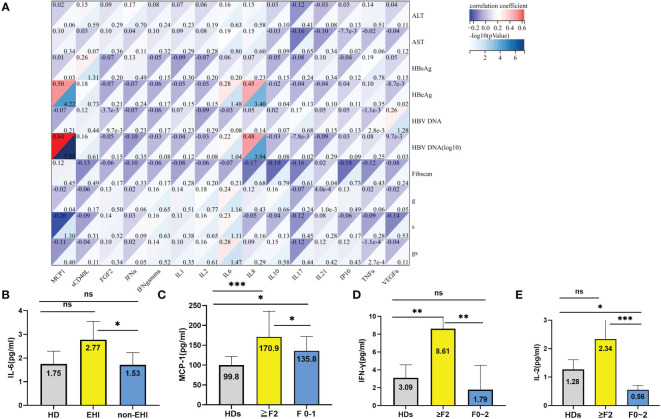
Relationship of the plasma cytokines with EHI. **(A)** Correlation of the cytokines with clinical characteristics and significant liver injury. **(B)** Comparison of the IL-6 levels in different groups. **(C)** Comparison of the MCP-1 levels in different groups. **(D)** Comparison of the IFN-γ levels in different groups; **(E)** Comparison of the IL-2 levels in different groups. *p <0.05, **p <0.01, ***p <0.001; ns, not significant.

Plasma cytokines, including sCD40 L, FGF-2, IFN-α, IL-1, IL-2, IL-6, IL-8, IL-10, IL-18, IL-21, IP-10, MCP-1, VEGF-α, TNF-α, and IFN-γ, were measured in 168 out of HBeAg-positive patients with HBV DNA >10^4^ IU/ml and 20 healthy controls to evaluate their clinical characteristics and EHI upon liver biopsy (**Figure 4A**). In immune-tolerant patients, when cytokine levels were included as independent variables, HBV DNA (log10) levels were positively correlated with MCP-1 and IL-8 (R = 0.64, p < 0.001; R = 0.48, p < 0.001), HBeAg levels were positively correlated with MCP-1 and IL-8 (R = 0.5, p < 0.001; R = 0.45, p < 0.001), and MCP-1 levels were negatively correlated with the histological grading of fibrosis (R = −0.26, p = 0.048) (**Figure 5A**).

The median levels of sCD40 L, FGF-2, IFN-α2, IL-1β, IL-8, IL-21, TNF-α, and VEGF-α in healthy donors were significantly higher than those in patients with chronic HBV infections and normal ALT levels. However, there was no significant difference between EHI and non-EHI (**Supplementary Table 2**). Only the IL-6 level was significantly higher in the EHI group than in the non-EHI group (2.77 *vs*. 1.53, Z = −13.32, p = 0.028) (**Figure 5B**). As all IT phase patients had a normal ALT level, the potential correlation between histological grading of fibrosis, which was staged from F0 to F4, and immune cytokines was analysed. We found that the MCP-1 level was significantly higher in patients with fibrosis stage ≥2 (≥F2) than in patients with F0–1 (170.89 ± 64.78 *vs*. 135.86 ± 36.05, F = 11.38, p = 0.04), and both were significantly higher than those in healthy donors (**Supplementary Table 2** and **Figure 5C**). Except for MCP-1, the IFN‐γ and IL-2 levels in the EHI group increased markedly (8.65 *vs.* 3.64 pg/ml, Z = 7.89, p = 0.011; 2.33 *vs.* 0.55 pg/ml, Z = 27.25, p = 0.001) (**Supplementary Table 3**, **Figures 5D, E**).

## Discussion

This study was based on a retrospective cohort of patients with chronic HBV infections and normal ALT levels (≤40 U/L) who underwent ultrasonography-guided percutaneous liver biopsy at Beijing You’an Hospital, Capital Medical University. Among these patients, 78.97% were over 30 years of age, 60.5% were men, and the overall proportion with EHI (≥G2 and/or ≥F2) was 50.1%. The proportion of chronic HBV infections in the GZ phase was 51.3%, and the EHI ratio in the GZ phase was 60.55% (63% in GZ-1 and 58.1% in GZ-2), which was significantly higher than that in the IT (39.8%) and IHC (37.7%) stages. This finding was consistent with the results of previous studies that pointed out the high proportion of patients in the GZ phase among 179 patients in the IT phase and 327 patients in the IHC phase (15). The EHI ratio of GZ accounted for 57.5%–81.8% and 51.6%–74.5% by liver biopsy in the two phases. A recent study of 3,366 patients with chronic HBV infections, including 38.7% in the GZ phase (10), reported a cumulative incidence of 4.6% developing hepatocellular carcinoma (HCC) within 10 years, which was 9.6 times that in IHC patients. This finding indicated that GZ patients had a high EHI ratio and a high risk for liver disease progression without antiviral therapy. Therefore, in the 2022 edition of the expert consensus (12) in China, antiviral therapy is recommended for patients in the GZ phase who are difficult to classify.

Furthermore, the 2022 edition of the expert consensus (12) in China has expanded the indications for antiviral therapy for chronic HBV infection. In particular, lowering the ALT treatment threshold (30 U/L for men and 19 U/L for women) and the requirement of being >30 years old will significantly increase the proportion of patients requiring treatment. In our study, 46.69% of patients met the new ALT treatment threshold, and their ratio of EHI was 56.2%. For IT and IHC phase patients, the new ALT thresholds reduced the EHI ratio to 32.6% and 37.8%, while GZ patients exceeding the ALT threshold had a significantly high EHI of 67.4% and 68.4%, respectively.

Several studies (16, 17) have shown that patients with high-normal ALT levels have a high risk for liver disease-related endpoint events. Kim et al. (18) showed that a high normal ALT level was associated with an increased risk of liver disease-related death: the odds ratio (OR) was 2.9 and 9.5 in patients with 20–29 and 30–39 U/L, respectively, compared to ALT <20 U/L. Montazeri et al. and Gobel et al. (19, 20) showed that compared to ALT <23 U/L (male) and <19 U/L (female), patients with ALT 23–46 U/L (male)/20–39 U/L (female) had significant EHI values (≥G2: 27% *vs*. 51%; ≥S2: 36% *vs*. 51%). Therefore, lowering the ALT threshold for antiviral therapy would result in a clinical benefit for more patients with chronic HBV infections, especially for those in the GZ phase.

Indeed, the EHI rate in this study was high due to the average age of all patients, with a median of 38.91 years old and 78.97% of patients >30 years old. In fact, great achievements in the safety of vaccination for newborns in China have been made, resulting in significantly decreased HBsAg prevalence rates in people aged 1–29 years (6). Currently, the vast majority of chronic HBV infections have already been established during the middle youth stage and have been included in the considerations for antiviral therapy.

In our study, IT phase patients were the youngest at 34.5 years, followed by GZ-1 patients at 37.63 years, while HBeAg-negative patients were significantly older at 40 and 43.5 years for IHC and GZ-2, respectively. Setting 30 years old as a threshold is critical for IT and IHC patients, but not for the GZ phase: EHI in GZ patients was the highest (55%–65%), irrespective of age, while EHI in IT and IHC increased significantly from 32.4% to 43.2% and 35.8% to 50% if the age threshold was >30 years, respectively. This is also consistent with the current guidelines that >30 years old is an independent risk factor for disease progression and can be an indication for initiating antiviral therapy. In a large US cohort study of 8,539 patients with chronic HBV infection followed up for 12 years, 317 developed HCC, age and family history were independent risk factors for HCC in patients without cirrhosis, and the OR was 32.9 (21). A study based on the Chinese Health Statistics Yearbook on the relative risk of HBV-related death suggested that the risk of HCC was significantly higher in patients >30 years old (22).

In particular, our findings suggest the potential for using the IL-6 level as a prognostic biomarker for EHI among patients with chronic HBV infections and normal ALT levels. IL-6 has also been implicated in HBV-induced hepatic necroinflammation (23). Furthermore, patients with chronic HBV infection and cirrhosis had significantly higher plasma IL-6 levels and more severe liver inflammation (24). Consistent with these reports, we observed that high IL-6 levels could indicate the severity of the inflammatory response and the risk of hepatic necrosis. MCP-1 attracts monocytes, T cells, and dendritic cells during infection, providing a sustained inflammatory bridge between innate and acquired immunity (25). In previous studies, MCP-1 levels were positively correlated with the viral load in HIV infection (26). One study (27) compared patients with chronic HBV infection and healthy individuals. The MCP-1 level was higher in chronic HBV infection, and a positive correlation of MCP-1 with HBV viral load and HBsAg level was discovered. In our study, the levels of HBeAg and HBV DNA were positively correlated with the levels of MCP-1, which was negatively correlated with the histological grading of fibrosis, implying the possible role of this chemokine in host immunity against viral replication. As mentioned previously, a higher level of MCP-1 is associated with breaking the balance and facilitating HBV immune tolerance. Further investigations are needed to clarify the underlying mechanism.

In addition, higher levels of serum IFN-γ and IL-2 were observed in patients with F ≥ 2. IFN‐γ can be secreted by CD8^+^ T cells and NK cells (28), and IL-2 production by HBV-specific CD4^+^ T cells (29) plays an important role in the efficient development of cytotoxic effector CD8^+^ T cells that contribute to the elimination of infection. These results may partly explain the pathogenesis of fibrosis progression as part of the immune response to chronic HBV infection, even within the immune-tolerant state with normal ALT levels.

The cytokine IL-8, also known as C-X-C motif ligand 8 (CXCL8), is a proinflammatory chemokine that acts as a chemoattractant of leukocytes (30). The correlation of viral load and HBeAg level with IL-8 showed a weak relationship in the HBV group, perhaps suggesting that high IL-8 favours the replication of the virus in chronic hepatitis B (31). Increased IL-8 was related to severe liver damage, and its levels decreased when these conditions improved in HBV (32). Additionally, this cytokine is considered to be one of those responsible for maintaining the inflammatory environment in HBV infection and was associated with HCC interfering with antitumour immunity (33).

Nevertheless, the present study has several limitations that affect the interpretation of our findings. This was a retrospective cross-sectional study that could not exclude selection bias. Moreover, the number of patients aged over 30 years was not large, and the follow-up of patients after the liver biopsy was insufficient.

The expansion of antiviral therapy indications may significantly increase the proportion of patients with chronic HBV infection receiving antiviral treatment. In our study, patients in the GZ phase, as well as IT and IHC patients older than 30 years, would indicate EHI. Lowering the antiviral therapy threshold for ALT was effective in screening out patients who already had EHI. Guided by the expansion of antiviral therapy indications, these patients could accept antiviral therapy in a timely manner, which might improve their long-term prognosis. In addition, our study showed that IL-6 levels were a good indicator of EHI even in the presence of an immune tolerance phase with normal ALT levels, and MCP-1 levels could reflect significant liver fibrosis at high levels of HBV DNA loads.

## Data availability statement

The original contributions presented in the study are included in the article/**Supplementary Material**. Further inquiries can be directed to the corresponding authors.

## Ethics statement

The study was approved by the ethics committee of Beijing Youan Hospital affifiliated with Capital Medical University ([2020]089). The patients/participants provided their written informed consent to participate in this study.

## Author contributions

SR and XC contributed to the conception of the study. WW contributed to cytokine detection with Luminex. KW and YZ contributed to the collection of the clinical data. SR and JL contributed significantly to the analysis and manuscript preparation. SR and LM performed the data analyses and wrote the manuscript. XC and SZ helped perform the analysis with constructive discussions. All authors contributed to the article and approved the submitted version.

## Funding

We are grateful to all the subjects who participated in this study. This work was supported by the Capital Health Development Scientific Research Project (2020-1-2181), Beijing Municipal Administration of Hospitals Clinical medicine Development of special funding support (ZYLX202125), National Science and Technology Key Project on “Major Infectious Diseases such as HIV/AIDS, Viral Hepatitis Prevention and Treatment”(2017ZX10302201-004, 2017ZX10202203-006), Capital Clinical Diagnostic Techniques and Translational Application Projects (Z211100002921059), You’an Foundation of Liver Disease and AIDS (YNKTQN20180209, BJYAYY-2020CX-01, BJYAYY-2020ZQN-01), Bethune Medical Science Research Foundation (SG095FN), and Beijing Hospitals Authority Youth Programme (QML20211702). The funder had no role in the study design, data collection and analysis, decision to publish, or preparation of the manuscript.

## Conflict of interest

The authors declare that the research was conducted in the absence of any commercial or financial relationships that could be construed as a potential conflict of interest.

The handling editor declared a shared parent affiliation with the authors at the time of review.

## Publisher’s note

All claims expressed in this article are solely those of the authors and do not necessarily represent those of their affiliated organizations, or those of the publisher, the editors and the reviewers. Any product that may be evaluated in this article, or claim that may be made by its manufacturer, is not guaranteed or endorsed by the publisher.
