# Supplementary material for: Effect of the change in antiviral therapy indication on identifying significant liver injury among chronic hepatitis B virus infections in the grey zone
Source: Front Immunol. 2022 Oct 27;13:1035923. doi: 10.3389/fimmu.2022.1035923 (PMC9647141; doi:10.3389/fimmu.2022.1035923)
Supplement: Supplementary file 1 [file Table_1.doc]

Supplementary Table 1. Univariate and multiple logistic regression analyses of factors associated with EHI

| Patients Parameter | EHI | Non-EHI | Univariate analyses | | | Multivariate | |
| --- | --- | --- | --- | --- | --- | --- | --- |
| **HBeAg-positive**  **(n=200)** | **n=101**  **(50.5%)** | **n=99 (49.5%)** | **P** | **OR** | **95%CI** | **OR** | **P** |
| Age(years) | 37.17±8.78 | 35.25±8.17 | 0.32 | 1.03 | 0.98-1.06 | 1.49 | 0.22 |
| Male(%) | 71(70.3%) | 57(57.6%) | 0.06 | 0.92 | 0.45-1.87 | 0.05 | 0.82 |
| ALT(U/L) | 29.92±10.42 | 26.78±8.67 | 0.02 | 1.02 | 0.98-1.06 | 0.93 | 0.33 |
| AST(U/L) | 26.65±15.36 | 21.84±9.62 | 0.01 | 1.01 | 0.97-1.04 | 0.43 | 0.51 |
| HBV DNA(log10 IU/ml) | 6.25±2.03 | 7.14±1.69 | 0.001 | 0.87 | 0.71-1.06 | 1.91 | 0.16 |
| **HBsAg(log10 IU/ml)** | **3.65±0.70** | **4.05±0.85** | **0.001** | **0.61** | **0.38-0.96** | **4.50** | **0.03** |
| HBeAg(COI) | 731±792 | 1238±900 | 0.001 | 1.00 | 0.99-1.00 | 0.84 | 0.36 |
| **Fibroscan(Kpa)** | **7.30±6.82** | **5.17±1.12** | **0.002** | **1.79** | **1.35-2.37** | **16.66** | **0.001** |
| **HBeAg-negative**  **(n=147)** | **n=73**  **(49.7%)** | **n=74 (50.3%)** | **P** | **OR** | **95%CI** | **OR** | **P** |
| Age(years) | 37.17±8.78 | 35.25±8.17 | 0.32 | 0.97 | 0.93-1.02 | 1.16 | 0.28 |
| Male(%) | 71(70.3%) | 57(57.6%) | 0.06 | 1.69 | 0.66-4.26 | 1.22 | 0.26 |
| ALT(U/L) | 29.92±10.42 | 26.78±8.67 | 0.02 | 1.06 | 0.92-1.21 | 0.65 | 0.42 |
| **AST(U/L)** | **26.65±15.36** | **21.84±9.62** | **0.01** | **1.07** | **1.01-1.13** | **5.88** | **0.02** |
| HBV DNA(log10 IU/ml) | 6.25±2.03 | 7.14±1.69 | 0.001 | 1.06 | 0.73-1.53 | 0.1 | 0.75 |
| **HBsAg(log10 IU/ml)** | **3.65±0.70** | **4.05±0.85** | **0.001** | **1.68** | **1.02-2.78** | **4.21** | **0.04** |
| **Fibroscan(Kpa)** | **7.30±6.82** | **5.17±1.12** | **0.002** | **2.36** | **1.58-3.54** | **17.45** | **0.001** |

Supplementary Table 2 Immunological Factors Associated with EHI

| **Factors**  **(pg/ml)** | **HDs**  **（n=20）** | **EHI**  **(n=80)** | **Non-EHI**  **（n=88）** | **P1(EHI vs Non-EHI)** | **P2(EHI vs HD)** | **P3(Non-EHI vs HD)** | **P** |
| --- | --- | --- | --- | --- | --- | --- | --- |
| sCD40L | 2942.6±1317.9 | 918.9±678.5 | 1015.1±866.7 | p=0.66  F=0.89 | P=0.000  F=21.31 | P=0.001  F=19.27 | P=0.000^*^  F=19.33 |
| FGF-2 | 182.2(124.8,301) | 100.7(24.1,1795) | 113.9(22.6,933.3) | P=0.53  Z=3.18 | P=0.002  Z=25.78 | P=0.002  Z=22.59 | P=0.004^&^  Z=11.05 |
| IFN-α2 | 66.4±22.9 | 37.9±20.9 | 38.3±20.5 | p=0.69  F=0.41 | P=0.006  F=6.78 | P=0.003  F=6.27 | P=0.003^*^  F=6.44 |
| IFN-γ | 3.1(1,4.6) | 1.2(0.6,79.1) | 1.2(0.6,18.4) | P=0.61  Z=-0.027 | P=0.01  Z=21.87 | P=0.004  Z=21.90 | P=0.013^&^  Z=8.65 |
| IL-1β | 23.4(3.2,41.0) | 8.9(0.9,347.8) | 9.1(1.2,270.5) | P=0.35  Z=-3.28 | P=0.06  Z=16.39 | P=0.012  Z=19.67 | P=0.042^&^  Z=6.21 |
| IL-2 | 1.4(0.5,1.7) | 0.4(0.1,16.8) | 0.4(0.2,3.0) | P=0.707  Z=-0.243 | P=0.000  Z=27.02 | P=0.001  Z=27.26 | P=0.001^&^  Z=13.3 |
| **IL-6** | **1.75(0.6,2.7)** | **2.77(0.8,10.3)** | **1.53(0.6,4.2)** | **P=0.036**  **Z=-13.32** | **P=0.07**  **Z=10.15** | **P=0.767**  **Z=1.15** | **P=0.03^&^**  **Z=6.95** |
| IL-8 | 17.9(3.1,78.0) | 1.5(0.4,6.3) | 1.6(0.4,5.4) | P=0.461  Z=-1.96 | P=0.000  Z=30.25 | P=0.000  Z=32.21 | P=0.000^&^  Z=17.69 |
| IL-10 | 1.1(0.6,2.9) | 0.9(0.2,88.7) | 0.9(0.2,4.6) | P=1  Z=-0.243 | P=0.001  Z=27.02 | P=0.002  Z=27.26 | P=0.47^&^  Z=1.50 |
| IL-17 | 184.9(6.8,1677) | 46.6(3.0,197.8) | 36.7(0.06,501.6) | P=1  Z=-1.23 | P=0.56  Z=0.89 | P=0.17  Z=1.45 | P=0.31^&^  Z=2.30 |
| IL-21 | 6.59±2.88 | 4.58±2.24 | 4.18±1.67 | p=0.466  F=0.54 | P=0.019  F=2.01 | P=0.006  F=2.41 | P=0.021^*^  F=4.11 |
| MCP-1 | 99.85±21.68 | 169.25±51.20 | 153.71±66.62 | p=0.122  F=1.54 | P=0.006  F=22.55 | P=0.000  F=69.39 | P=0.014^*^  F=4.61 |
| TNF-α | 24.69±9.05 | 14.08±7.21 | 14.51±8.00 | p=0.568  F=-0.57 | P=0.006  F=2.72 | P=0.002  F=3.07 | P=0.008^*^  F=9.73 |
| VEGF-α | 89.6(14.9,289.4) | 23.1(0.6,136.1) | 27.2(0.4,179.5) | P=0.552  Z=-3.013 | P=0.028  Z=2.204 | P=0.01  Z=2.587 | P=0.034^&^  Z=6.77 |
| IP-10 | 115.6(45.0,248.9) | 136.2(52.3,4160) | 134.4(49.8,411.5) | P=0.89  Z=-1.243 | P=0.58  Z=2.02 | P=0.99  Z=-1.26 | P=0.331^&^  Z=2.207 |

Histological grading of liver inflammation is classified into G0 ~G4, and fibrosis was staged from S0 to S4. significant liver histological injury (EHI) were defined as inflammation grade≧2 (≧G2) and/or fibrosis stage≧2(≧S2).Data are presented as mean±SD or median (IQR); P < .05: statistically significant; * LSD was used for homogeneity of variance;# Tamhane's T2 method was used for heterogeneity of variance; & Kruskal-Wallis rank sum test was used for non-normal distribution;P1: Comparison between EHI group and Non-EHI group; P2, Comparison between EHI group and HD group; P3, Comparison between Non-EHI group and HD group; P, Comparison of the patients in three groups.

Supplementary Table 3 Immunological Factors Associated with fibrosis stage≧2(≧S2)

| **Factors**  **(pg/ml)** | **HDs**  **（n=20）** | **Patients with fibrosis stage≧2(≧F2)(n=49)** | **Patients with fibrosis stage＜2(＜F2)(n=119)** | **P1(****≧F2 vs ＜F2)** | **P2(≧F2 vs HD)** | **P3(＜F2 vs HD)** | **P** |
| --- | --- | --- | --- | --- | --- | --- | --- |
| sCD40L | 2942.6±1317.9 | 906±661.9 | 986.6±810.7 | p=0.74  F=2.89 | P=0.000  F=41.21 | P=0.000  F=36.11 | P=0.000^*^  F=19.26 |
| FGF-2 | 182.2(124.8,301) | 97.9(31.34,1795) | 112.4(22.6,933.3) | P=1  Z=-0.58 | P=0.015  Z=23.88 | P=0.004  Z=24.46 | P=0.005^&^  Z=10.68 |
| IFN-α2 | 66.4±22.9 | 38.8±24.4 | 37.8±19.1 | p=0.855  F=1.10 | P=0.003  F=7.99 | P=0.001  F=8.07 | P=0.003^*^  F=6.46 |
| **IFN-γ** | **3.09±1.47** | **8.65±6.57** | **3.64±2.15** | **p=0.011**  **F=7.89** | **P=0.01**  **F=6.21** | **P=0.17**  **F=1.11** | **P=0.03^*^**  **F=3.76** |
| IL-1β | 23.4(3.2,41.0) | 8.9(0.9,347.8) | 8.9(1.2,270.5) | P=1  Z=-3.8 | P=0.212  Z=15.19 | P=0.038  Z=19.0 | P=0.04^&^  Z=6.25 |
| **IL-2** | **1.27(0.5,1.7)** | **2.33(0.1,16.8)** | **0.55(0.2,3.0)** | **P=0.001**  **Z=27.25** | **P=0.08**  **Z=3.88** | **P=0.06**  **Z=3.28** | **P=0.001^&^**  **Z=13.3** |
| IL-6 | 1.9(0.6,2.7) | 1.8(0.9,10.3) | 1.3(0.6,7.5) | P=0.77  Z=-2.32 | P=1  Z=-3.17 | P=0.6  Z=1.15 | P=0.43^&^  Z=1.67 |
| IL-8 | 17.9(3.1,78.0) | 1.3(0.4,4.8) | 1.7(0.4,6.3) | P=0.39  Z=4.77 | P=0.000  Z=29.84 | P=0.000  Z=34.58 | P=0.000^&^  Z=18.26 |
| IL-10 | 1.1(0.6,2.9) | 0.7(0.2,9.2) | 0.9(0.2,88.7) | P=1  Z=-1.243 | P=0.45  Z=4.02 | P=0.28  Z=3.26 | P=0.40^&^  Z=1.94 |
| IL-18 | 184.9(6.8,1677) | 62.2(14.1,197.8) | 35.78(0.06,501.6) | P=1  Z=-0.73 | P=0.56  Z=1.19 | P=0.28  Z=2.45 | P=0.098^&^  Z=4.643 |
| IL-22 | 6.59±2.88 | 4.46±2.13 | 4.36±1.95 | p=0.877  F=-0.09 | P=0.02  F=2.13 | P=0.008  F=2.22 | P=0.027^*^  F=3.823 |
| **MCP-1** | **99.85±21.68** | **170.89±64.78** | **135.86±36.05** | **p=0.04**  **F=11.38** | **P=0.000**  **F=28.23** | **P=0.02**  **F=15.33** | **P=0.000^*^**  **F=16.01** |
| TNF-α | 24.69±9.05 | 12.48±6.14 | 15.00±7.96 | p=0.258  F=1.21 | P=0.000  F=12.21 | P=0.002  F=9.21 | P=0.002^*^  F=7.12 |
| VEGF-α | 89.6(14.9,289.4) | 23.1(0.6,136.1) | 23.4(0.4,179.5) | P=1  Z=-2.31 | P=0.11  Z=17.68 | P=0.026  Z=19.993 | P=0.031^&^  Z=6.92 |
| IP-10 | 115.6(45.0,248.9) | 129.4(72.6,214) | 136.1(49.8,4160) | P=0.68  Z=-0.243 | P=0.72  Z=0.82 | P=0.21  Z=-1.26 | P=0.323^&^  Z=2.261 |

Histological grading of fibrosis was staged from F0 to F4. Data are presented as mean±SD or median (IQR); P < .05: statistically significant; * LSD was used for homogeneity of variance;# Tamhane's T2 method was used for heterogeneity of variance; & Kruskal-Wallis rank sum test was used for non-normal distribution;P1: Comparison between≧F2 and＜F2; P2, Comparison between≧F2 and HD group; P3, Comparison between＜F2 group and HD group; P, Comparison of the patients in three groups.
